# Participants in Transcription–Replication Conflict and Their Role in Formation and Resolution of R-Loops

**DOI:** 10.3390/ijms26146951

**Published:** 2025-07-19

**Authors:** Anastasiia T. Davletgildeeva, Nikita A. Kuznetsov

**Affiliations:** 1 Institute of Chemical Biology and Fundamental Medicine, Siberian Branch of Russian Academy of Sciences, Novosibirsk 630090, Russia; nikita.kuznetsov@1bio.ru; 2 Department of Natural Sciences, Novosibirsk State University, Novosibirsk 630090, Russia

**Keywords:** genomic instability, transcription–replication conflict, transcriptional pausing, replication fork stalling, non-B DNA structure, R-loop, enzymatic activity, processivity, enzyme kinetics

## Abstract

The DNA of all living organisms is a common matrix for both replication and transcription processes. This sometimes leads to inevitable collisions between DNA replication and transcription machinery. There is plethora of evidence demonstrating that such collisions (or TRCs) are one of the most common and significant reasons for genomic instability. One of the key outcomes of TRCs is the accumulation of non-canonical DNA secondary structures, including R-loops. R-loops are three-stranded DNA–RNA hybrids with a displaced third single-stranded DNA fragment. Although R-loops are thought to play several functional roles in biological processes, an imbalance in their metabolism has been proven to have severe consequences. In this review, we attempt to summarize the current knowledge of the participants in the process of R-loop regulation in cells, with an emphasis on eukaryotic systems. We also touch upon the conditions favoring TRCs and the possible ways of dealing with these conflicts.

## 1. Introduction

The effective firing of replication origins and the ongoing progression of the replication fork play crucial roles in the genome duplication process, which is essential to every living cell. This process is important for normal cell cycle progression and to avoid deleterious DNA damage and cell death [1]. Nevertheless, the progression of the replication fork can be disrupted if it meets unrepaired DNA lesions, non-canonical (non-B) DNA secondary structures, or various nonhistone proteins that are bound to DNA [2]. If the replication fork stalls, replication checkpoint factors promote its stabilization to ensure the association of the replisome with the template and allow for the possibility of resuming the replication process, as its impairment is a major issue for dividing cells [3].

Considering the fact that DNA in a cell is not only a substrate for replication, but also for transcription, it is natural that the interference of these two processes, which are localized at the same genome region, is a prominent example of an endogenous threat to optimal replication progression [4,5]. A rather common and telling fact indicating that transcription is a source of replicative stress is that the active transcription process leads to an increase in spontaneous mitotic recombination. It is widely accepted that the passage of transcription leads to the most detrimental DNA lesions, double-strand breaks (DSBs), which are repaired via recombination [6]. Indeed, during transcription, the positive supercoiling of processed DNA occurs before this process; however, after the RNA polymerase (RNAP) has passed through, DNA with negative supercoiling remains [2,7]. These topological changes in DNA can accumulate in regions of highly transcribed genes, disrupting any further passage of the replication fork through these regions [2,8]. Topoisomerase I (Top1) is the main enzyme responsible for the resolution of negative supercoiled DNA associated with transcription [9]. In contrast, topoisomerase II (Top2) is needed to relax the positive supercoils that are concentrated before the elongating RNAP [10]. When the activities of these two enzymes are abolished, highly transcribed regions of DNA tend to accumulate torsional stress, which can subsequently lead to stretches of single-stranded DNA (ssDNA) behind the RNAP [11,12]. The resulting persistence of ssDNA regions may lead to a burst in DNA damage levels due to the higher susceptibility of such regions to genotoxic agents, or it could give rise to the formation of different non-B DNA structures [2].

DNA molecules are capable of adopting several various conformational forms besides the classic B structure, including hairpins, left-handed Z-DNA, G-quadruplexes, triplex DNA (or H-DNA), and RNA–DNA hybrids. Negative supercoiling and the unwinding of the DNA duplex that accompany replication or transcription processes ensure the formation of such structures along with a suitable DNA sequence [2]. A number of the negative effects of such non-B structures on the genome’s stability are due to their interference with replication fork passage [3,13]. Additionally, the occurrence of these structures within gene bodies can provoke RNAP stalling and the termination of transcription elongation [14]. RNAP stalling itself acts as a signal for transcription-coupled nucleotide excision repair (TC-NER) in cells. Thus, although nucleotide excision repair (NER) usually proceeds with low error levels, the enrollment of the ssDNA intermediate, which is sensitive to damaging agents, and gap-filling DNA synthesis could be a path for introducing new errors [2,15].

**Figure 1:**
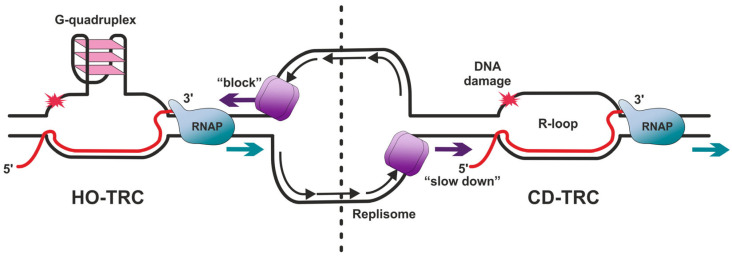
Types of TRCs and R-loop formation. HO-TRC (**left**) is head-on transcription–replication conflict, while CD-TRC (**right**) is co-directional transcription–replication conflict. RNAP is RNA polymerase. Replisome was drawn schematically based on [30].

Transcription–replication collisions or conflicts (TRCs) are considered to be one of the major sources of the replication stress associated with transcriptional dysregulation [16]. The type, and consequently severity and harmfulness of TRCs depends on the direction in which the collision of transcription and replication processes occurs. When the replication fork moves in the same direction as the transcription machinery and they use the same DNA strand as a template, such a conflict is called co-directional TRC (CD-TRC, Figure 1), which is considered to occur at a lower cost to the cell and be more easily resolved [17]. Since the velocity of the passage of *E. coli* replication forks is 10 times higher than the transcription machinery, their co-directional collision is inevitable [18]. Nevertheless, bacterial replication forks were demonstrated in vitro to be able to pass RNAP without displacing it and a transcript during CD-TRCs [19,20]. When replication and transcription processes occur in opposite directions and collide with each other, the conflict is called a head-on TRC (HO-TRC, Figure 1) [16,21,22], and the detrimental consequences of this type of TRC have long been recognized [23]. It was demonstrated that DNA polymerase is unable to bypass RNAP and is forced to pause during HO-TRCs [23], while it is only slowed down during CD-TRCs [24]. However, RNAPs appear to be able to resume RNA synthesis in either type of TRC. Although it is often forced to dissemble during TRC resolution, replication restart proteins, which have been found to be essential in many procaryotes, rebuild the disrupted replisome at TRC sites [25]. In bacterial systems, the replisome has been shown to be able to displace RNAP from DNA in both CD-TRCs and HO-TRCs, although in the latter case, it requires the help of the elongation factor Mfd [26,27]. This translocase protein, which is involved in NER, recognizes stalled RNAPs and displaces them from DNA in an ATP-dependent manner [28,29].

## 2. Dealing with TRCs

The fact that transcription tends to be a constant threat to the normal progression of replication, especially when these two processes collide, suggests that there must be various factors and mechanisms that promote the progression of a stalled replication fork and maintain genomic stability in such cases [2].

One of the general characteristics that indicates a greater level of danger from HO-TRCs for the genome’s stability is the prevailing co-directional orientation of actively transcribed genes. Indeed, bacterial genomes were demonstrated to be mostly organized to orient vital and highly transcribed genes in the same direction as replication, although it necessarily favors evaluation during CD-TRC events [31]. Numerous studies have demonstrated the same overall orientation of the genome for eukaryotes as well [32,33,34,35,36]. Thus, it has been shown that the transcription start sites of many long and highly transcribed genes coincide with replication initiation sites, while the transcription end sites of these genes often overlap with their replication termination sites [34,37,38]. Among the most problematic for the eucaryotic replication of actively transcribed genes are ribosomal DNAs (rDNAs). About 150 rDNA repeats are located at the *S. cerevisiae* rDNA locus, each consisting of 35S (RNAP I–transcribed) and 5S (RNAP III–transcribed) pre-rRNA genes [39,40]. These loci are also equipped with special sites that act as barriers to the passage of the replication fork downstream of the 35S gene [41] due to the binding of particular proteins to them, such as Fob1 known to create a unidirectional block to replication forks [42]. This allows for the blocking of the active passage of the replication fork at certain sites by slowing it down, thus avoiding the occurrence of HO-TRC events [43].

At the same time, there might be a reason for the existence of head-on-oriented genes. It has been suggested that head-on genes must undergo elevated rates of TRC-induced mutagenesis, so it is possible that this orientation does not establish by chance and that selection is aimed at achieving high variability in these genes [44,45]. CD-TRCs are able to provoke the disruption of replication at certain sites and even cause DSB emergence [46,47]. In eukaryotes, it was demonstrated that HO-TRCs at RNAP II-transcribed genes can induce recombination [48]. Moreover, transcription-induced stress, especially in the case of Top1 dysfunction, can provoke the elevation and recombination of S-phase-dependent DNA breaks [49,50]. As long as the transcription of long genes can take several cell cycles, avoiding TRCs at such genes can be quite difficult, which is further complicated by the fact that some long genes may overlap with hotspots for chromosomal instability (or so-called common fragile sites), which replicate late in the S-phase [51,52].

Topological stress leads to HO-TRCs causing more harm to cells than CO-TRCs, meaning that topoisomerases play a key role in HO-TRC-induced stress prevention. It was demonstrated by Lang and Meriikh that the prevention of HO-TRCs specifically requires the relaxation of positive supercoiling by type II topoisomerases DNA gyrase and Topo IV in *Bacillus subtilis.* Moreover, it was shown that DNA gyrase and Topo IV preferentially associate with head-on-oriented genes. At the same time, it seems that the subsequential introduction of negative supercoiling by DNA gyrase is responsible for the formation of R-loops [53]. An increase in the rate of positive supercoiling of DNA resulting from HO-TRCs was found to stall the progress of transcription and replication machinery [5,53,54,55].

The results of several in vitro studies demonstrate that the blockade of the replisome resulting from HO-TRC events does not necessarily lead to its disruption, and replication can be resumed in the presence of certain helping factors, such as T4 Dda helicase and Mfd translocase [23,26]. To displace proteins interfering with the advancement of the replication fork, *E. coli* utilize accessory helicases, such as Rep, UvrD, and DinG [56], while for *B. subtilis* helicase, PcrA was demonstrated to assist replication forks with progressing through several genes placed in a head-on orientation to replication [57]. The recruitment of various accessory DNA helicases to resolve the progression of replication forks has also been demonstrated in eukaryotic cells. One of the most-studied examples is the helicase Rrm3 of *S. cerevisiae*, which is a part of the replisome complex and was demonstrated to facilitate the passage of the replication fork through the protein-bound regions of DNA [58,59].

## 3. TRC-Induced Non-B DNA Structures

### 3.1. G-Quadruplexes

G-quadruplexes are the result of the folding of the G-rich DNA motifs into tetraplex structures, with stacked groups of the four guanines being stabilized in one planar orientation via Hoogsteen hydrogen bond pairing [60] (Figure 1). The appearance of G-quadruplexes on the replication or transcription pathway can become a serious obstacle to the passage of the replication forks or RNAPs [61,62]. Indeed, it is known that the promoter regions and 5′-untranslated regions of highly transcribed DNA genes are enriched in sequences that are prone to forming G-quadruplex structures [63]. For example, eukaryotic rDNA consisting of a series of repeating pre-rRNA genes is predicted to have high potency in non-B structure formation, specifically G-quadruplexes [64,65]. Several pieces of evidence were found to indicate that specialized helicases resolving G-quadruplexes participate in the facilitation of replication and transcription [66,67,68]. Thus, the highly conserved fission yeast Pfh1 helicase, which is homologous to *S. cerevisiae* Rrm3 [69,70], was demonstrated to have a preference for binding rDNA and being able to unwind model rDNA G-quadruplex structures [71]. The ability to unwind G-quadruplex structures has been demonstrated for several human helicases as well, such as the Bloom syndrome protein (BLM), Werner syndrome helicase (WRN), FANCJ, RTEL1, and PIF1; however, most of these experiments were conducted in vitro, so the real role of these enzymes in facilitating replication passage through G-quadruplex obstacles remains to be unraveled [67]. For example, it was demonstrated by Isik et al. that FANCJ helicase participates in the restart of R-loops–stalled forks though its interaction with the mismatch repair (MMR) factors MutSβ and MLH1, components of the MutLβ complex and several other complexes participating in MMR [8].

In addition to the specialized helicases that are capable of resolving G-quadruplex structures along the replication fork passage, polymerases that carry out trans-lesion synthesis (TLS) are also capable of dealing with such obstacles in the cell [60]. DNA replication machinery can utilize DNA-damage-tolerant TLS to bypass some lesions, and thus avoid malicious replication stops. It has been demonstrated that this mechanism can be carried out in eukaryotic cells to pass through G-quadruplex regions during replication; in this case, TLS is achieved by the work of the polymerases Rev1 [72], η [73,74], κ [75], and θ [76].

### 3.2. R-Loops

The first demonstration of co-transcriptional R-loops being a threat to genomic stability through recombination intensification was the emergence of the hyper-recombination phenotype of the *S. cerevisiae* mutants of the THO/TREX, a conserved eukaryotic protein complex which plays important role in transcription and mRNA metabolism [77,78,79]. It was shown that this phenotype also depends on the capacity of the nascent RNA molecule to form an R-loop behind the elongating DNA polymerase [77].

R-loops are structures consisting of a DNA–RNA hybrid and the displaced ssDNA (Figure 1) and have several different ways of forming in cells, including TRCs [80,81,82,83]. R-loops were first discovered in bacteria and were shown to initiate DNA replication [84]. Today, these structures are known to exist in the cells of many living organisms, including mammals, and they participate in a variety of processes, including DNA replication, the regulation of gene expression, and immunoglobulin class-switch recombination; however, they also pose a serious threat to genomic stability [82,85,86,87]. Given that R-loops appear to have great functional significance in certain conditions, they are usually divided into physiological and pathological, that is, those that are formed during specialized processes and impacted by specific factors, and those that are formed spontaneously or by mistake and cause disturbances in the genetic stability of organisms [88]. It was suggested to separate RNAPII-promoted R-loops into two classes: (1) promotor-paused R-loops, which are short R-loops that form at a high frequency during promoter–proximal pausing by RNA polymerase; and (2) the considerably less common long elongation-associated R-loops, which occur throughout gene bodies [89]. The authors further suggested that Class 1 R-loops are responsible for most cases of R-loop-induced genome instability.

Persistent R-loops are one of the reasons for ongoing DNA damage and transcription-associated replication stress. The formation of R-loops can lead to DNA damage and genetic instability in several ways: First, the existence of very long R-loops leads to the formation of extended ssDNA regions, which are more sensitive to the occurrence of various forms of damage than double-stranded DNA (dsDNA). This is effectively demonstrated by the fact that in *E. coli*, the DNA strand that is not currently transcribed (the ssDNA fragment in the R-loop) is more often a source of mutation during transcription than the transcribed one (the DNA that is hybridized with RNA) [90]. Second, it was shown that reducing the factors participating in the prevention of R-loop formation or their resolution leads to the active transformation of these structures into DNA DSBs by the NER endonucleases XPF (ERCC1) and XPG (ERCC5), which are named after the rare autosomal recessive congenital syndrome xeroderma pigmentosum (XP) [91]. Third, the emergence of R-loops, as well as other secondary DNA structures and DNA–protein complexes, along the pathway of RNAPs can lead to transcription arrest [92]. Finally, the emergence of persistent R-loops in the replication machinery passage may end up blocking its progression and causing the replication fork to collapse, leading to subsequent DSB formation [85,93]. HO-TRC-induced R-loops may lead to replication fork reversal (the conversion of replication forks into four-way junctions via strand exchange reactions). This process involves replisome disruption and the protection of the newly formed DNA duplex with RAD51 filaments, stabilized by breast cancer type 2 susceptibility gene protein (BRCA2) [94,95,96]. Despite the fact that in general replication fork reversal is a protective cellular mechanism, it can have detrimental consequences if not properly regulated. Reversal that is not properly resolved, or excessive fork reversal can result in DNA degradation by nucleases, leading to DNA break emergence, genomic instability, and potentially cell death [97,98,99].

In fact, the details of what happens when the replication fork encounters R-loops remain murky. In the case of eukaryotes, this is largely due to the plasticity of their replicative apparatus, which complicates the study of the influence of R-loops on the possibility of resolving TRCs. In their work, Hamper et al. developed an episomal system to study TRCs and demonstrated the main difference between the cellular responses toward CD- and HO-TRC-induced R-loops [30]. First, the level of R-loop accumulation itself was shown to be dependent on the type of TRC, with HO-TRCs leading to R-loop formation and CO-TRCs resulting in R-loops being resolved [30]. It also appeared that CD-TRCs induce serine protein kinase ATM activation, while HO-TRCs are ATR-inducing. Ataxia telangiectasia-mutated (ATM) and RAD3-related DNA damage response (DDR) kinases (ATRs) are typically activated in response to stretches of replication protein A (RPA)-coated ssDNA at stalled replication fork sites, while ATM is mostly activated by DSBs [100]. The recent work by Zhand et al. linked the activation of ATR by HO-TRC-induced R-loops to the RNA-editing enzyme ADAR1 and its interaction with TOPBP1 on R-loops. It was also assumed that ADAR1 further recruits specialized DHX9 and DDX21 helicases to unwind R-loops [101].

#### 3.2.1. Factors Promoting R-Loop Formation

Despite the fact that R-loops are ubiquitously formed in the cells of living organisms, little is known about the factors promoting their formation. Generally, it is thought that R-loops are mostly formed co-transcriptionally (in a *cis* manner) due to the hybridization of the newly synthetized nascent messenger RNA and the DNA template [85,102]. There is also a possibility of R-loops being formed in a *trans* manner when RNA hybridizes with a complementary DNA sequence far away from the original site of its transcription [103]; recombination factors, such as RAD51 and RAD52, as well as the RAD51 bacterial homolog RecA, may play a role in this *trans* R-loop formation [87,104].

There is presumably a direct link between the formation of G-quadruplex structures and R-loops. R-loops are formed at the promoter and terminator sites of genes [105,106,107] and are particularly common where there is an imbalance between the concentrations of C and G on complementary strands [105,108]. Such sites are known to be prone to the formation of G-quadruplex structures, while it has also been shown that the transcription of G-rich sites does indeed lead to the accumulation of R-loops [83,109,110]. For example, it was demonstrated that the helicase DDX1 is able to convert G-quadruplexes into R-loops, which promotes IgH class-switch recombination [111]. Apparently, the most significant impacts on the cell are caused by TRCs causing RNAP arrest, since this can lead to the blockage of the replication fork and a collision with moving RNAP, as it does not necessarily result in the stalling of the replication machinery [112,113]. As was mentioned above, the occurrence of different non-B DNA structures, including R-loops, before the replisome passage can easily result in RNAP stalling [14]. On the other hand, the extended pausing of RNAP on transcription sites can stabilize R-loops [114]. It is therefore hard to distinguish between the harmful effects of R-loops and RNAPs and the detrimental consequences of HO-TRCs. In *Drosophila melanogaster,* RNAP II abundance appears to be a more significant R-loop-inducing factor than even the properties of R-loop-forming DNA (GC-rich sequences). Global R-loop induction was demonstrated to be strongly coupled with RNAP II pausing [115]. It was also shown that RNAP II tends to accumulate with R-loops at HO-TRC sites and acts as the main obstacle to replication fork progression [116].

In a comprehensive study [117], more than 800 proteins that bind to DNA–RNA hybrids (particularly in R-loop structures) were identified, and more than 300 of these preferred hybrid structures to dsDNA, including known R-loops interactors, such as RNaseH 1 and DDX5. Such studies could provide valuable insights to help identify which of these proteins play important roles in both R-loop formation and resolution. In this review, we have tried to compile the available information on certain protein factors that supposably promote R-loop formation, which are all listed in Table 1.

In their study [114], Chakraborty et al. drew a line between R-loop formation and the role of certain splicing factors. Apparently, DEXH-box RNA helicase DHX9 (also known as RNA helicase A, RHA), which is known to participate in the assembly of splicing factors onto nascent RNA, promotes the formation of R-loops in cells where splicing factors are absent. On the other hand, in other studies, the ability of DHX9 to unwind DNA–RNA hybrids and G-quadruplex structures has instead been shown to be associated with its putative role in preventing the formation of R-loops [118,119]. It is possible that specialized helicases’ activity may have opposite consequences for R-loop persistence under different conditions. Their possible role in controlling DHX9 action might be facilitated by the factors attracting these helicases to R-loops, such as polycytosine (poly(C))-binding protein 1 (PCBP1), which was demonstrated to modulate transcription by regulating the accumulation and activity of DHX9 [120]. It was also shown that DHX9 is phosphorylated at Ser321 by HO-TRC-activated ATR, which facilitates the interaction of this helicase with BRCA1 and RPA and causes its association with R-loop sites [121].

**Table 1:** Protein factors promoting R-loop formation.

Protein	Protein Function (Known Role in The Organism)	Possible Role in R-Loops Formation	Ref.
RAD51 (RecA *)	Plays an important role in homologous strand exchange, a key step in DNA repair, through homologous recombination (HR). Coats DNA-forming nucleoprotein filaments. Interacts with many partners. Participates in the Fanconi anemia (FA) pathway.	Possibly involved in the formation of *trans* R-loops.	[87,104]
**RAD52**	Involved in DSB repair. Plays a key role in genetic recombination and DNA repair, promoting the annealing of complementary single-stranded DNA and stimulating RAD51 recombinase.		
**RNAP II (RNAP)**	DNA-dependent RNA polymerase which synthesizes mRNA precursors and several functional non-coding RNAs.	Tends to accumulate together with R-loops at HO-TRC sites and acts as a main obstacle to replication fork progression. Extended pausing can stabilize R-loops. RNAP was demonstrated to partially protect short R-loop segments from RNase H1-facilitated cleavage.	[114,116,122]
**DHX9** (also known as **RHA**)	RNA helicase A. Participates in the assembly of splicing factors onto nascent RNA.	Promotes the formation of R-loops in cells where splicing factors are absent.	[123]
**PRC2**	Polycomb repressive complex. Exhibits histone methyltransferase activity and primarily methylates histone H3 on lysine 27.	Opens DNA bubbles and induces the formation of RNA–DNA hybrids.	[124]
**RPA**	Binds and stabilizes ssDNA intermediates that form during DNA replication or upon DNA stress.	Supposably promotes R-loop formation by binding to RNA.	[125]
**TET1**	DNA dioxygenase. Catalyzes oxidation of epigenetic 5-methylcytosine to 5-hydroxymethylcytosine.	Catalytic activity in the region of transcribed genes leads to preferential formation of R-loops therein.	[86,126]

* Procaryotic homolog.

In their recent work, Alecki et al. demonstrated that *D. melanogaster* Polycomb repressive complex 2 (PRC2) opens DNA bubbles and induces the formation of RNA–DNA hybrids, which are essential components of R-loops [124]. This conclusion was supported by the observation that the activity of RING1B, a core component of PRC1, is crucial for the formation of nascent RNA transcripts and R-loops at the estrogen receptor alpha (Erα) target genes [127]. In contrast, Sanchez et al. demonstrated that the human Polycomb group proteins BMI1 and RNF2 suppressed TRCs, presumably through the regulation of RNAP II elongation, thus protecting common fragile sites from breakage [128].

It is interesting that the role of RPA, which is generally considered to preferentially bind ssDNA, in R-loop formation was also suggested. It was shown that RPA is not only able to bind RNA with considerably high efficiency, but also tends to associate with R-loops in vivo [125]. The authors of this work suggest that RPA promotes R-loop formation by binding to RNA. Interestingly, Mazina et al. demonstrated the ability of human DNA polymerases to initiate DNA synthesis by utilizing RPA-generated R-loops, thus reproducing replication restart in vivo. Moreover, it was suggested that RPA, localizing at the R-loop sites in SETX-deficient cells, prevents R-loop-induced DSB formation [129].

An interesting link between DNA methylation and R-loops has been demonstrated in several studies, suggesting new protein candidates for promoting R-loop formation. It was demonstrated that TET1, a member of the ten-eleven translocation (TET) DNA dioxygenases family, can be recruited to CpG island promoters through interactions with growth arrest and DNA damage protein 45A (GADD45A), which specifically binds to R-loops [86]. Sabino et al. demonstrated that the emergence of 5-methylcytosine (m^5^C)—which is subsequently transformed into 5-hydroxymethylcytosine (hm^5^C) via the dioxygenase activity of TET—in the body of transcribed genes favors R-loop formation [126]. Moreover, the depletion of TET in cells leads to a decrease in R-loop levels. TET DNA dioxygenases are key participants in the modulation of epigenetic methylation levels in DNA [130,131,132]. Another essential activity in this process is carried out by the enzymes belonging to the DNA methyltransferase family (DNMT) [133,134,135]. In their recent work, Shih et al. discovered that abolishing DNMT3B activity in cells leads to an increase in the XPG/XPF-mediated conversion of R-loops into DSBs [136]. The authors suggested that the de novo methyltransferase DNMT3B somehow protects R-loops from elevated processing by XPG and XPF. These observations reveal new facets of the relationship between R-loop-induced replication stress and the epigenetic modulation of transcription.

#### 3.2.2. Factors Facilitating R-Loop Suppression

**Figure 2:**
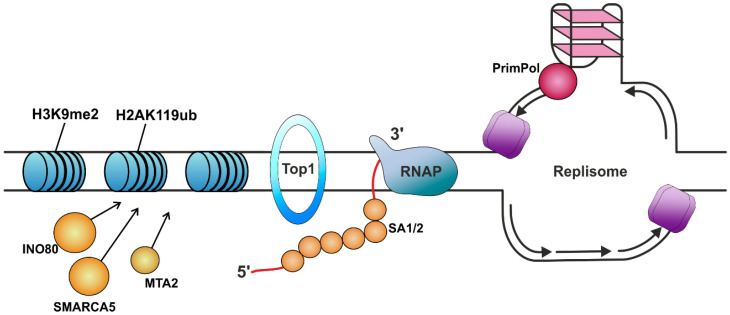
The factors preventing R-loop formation. RNAP is RNA polymerase. Primase-Polymerase (PrimPol) participates in the replication process of sequences that are prone to R-loop formation. The coating proteins cohesin SA1/2 supposably participate in the protection of newly synthesized RNA. Topoisomerase Top1 resolves the accumulation of local negative supercoils on transcribed regions. The chromatin structure, including marks such as H3K9 dimethylation (H3K9me2) and H2AK119 ubiquitination (H2AK119ub) and the enrolment of chromatin remodeling factors (INO80, SMARCA5, and MTA2), also plays a role in preventing R-loop formation.

**Figure 3:**
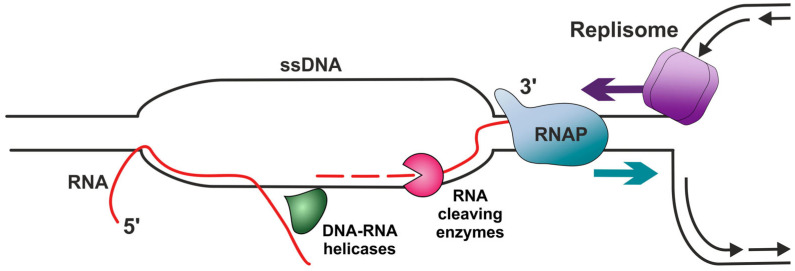
Factors directly resolving R-loops. RNA-cleaving enzymes include RNase H1 and H2, ribonuclease DICER, RNA exonuclease 5 (REXO5), and flap endonuclease 1 (FEN1). Examples of DNA-RNA helicases include senataxin (SETX), members of RecQ-like family helicases (Bloom syndrome protein (BLM), Werner syndrome helicase (WRN), and RECQ5) and DEAD/DEXH-box RNA helicases (such as DDX1, DDX5, DDX19, DDX21, DDX37, DDX50, and DHX9).

**Figure 4:**
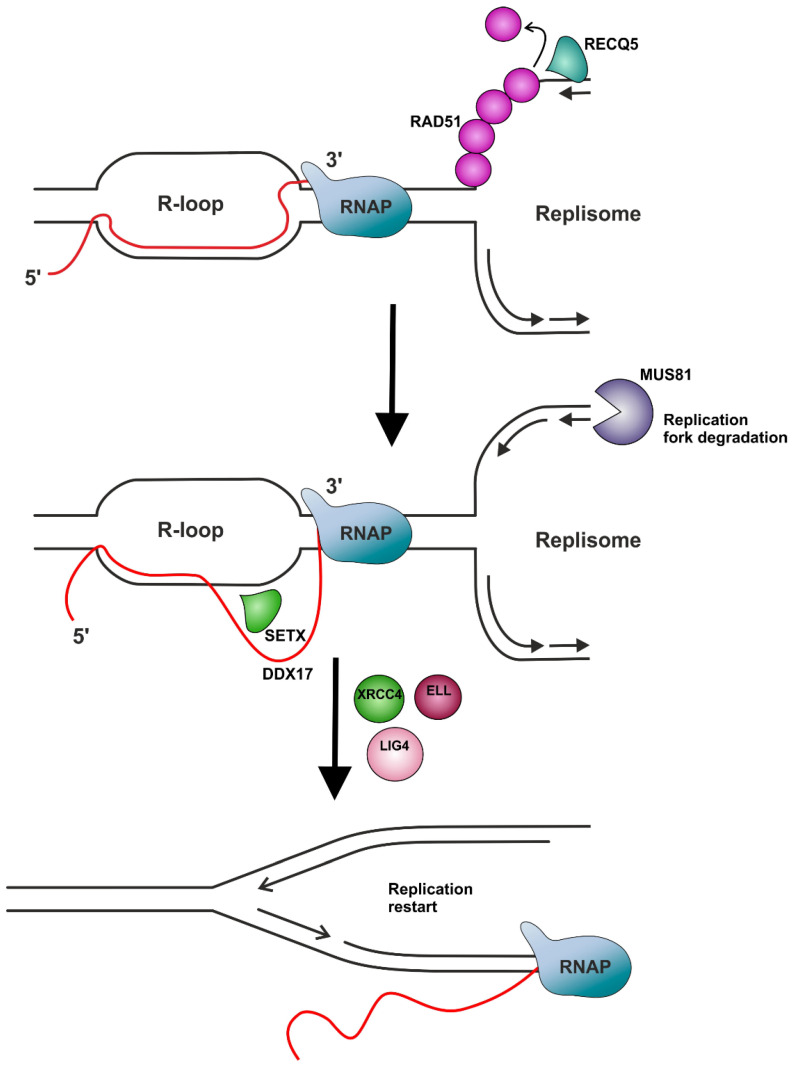
The MUS81-dependent mechanism of resolving R-loops. The helicase REQ5 removes RAD51 from the stalled replication fork to facilitate MUS81 endonuclease’s cleavage of the leading chain of the stalled replication fork. The helicase SETX or DDX17 directly unwinds the R-loop. The further religation of the fork is facilitated through the participation of the DNA ligase IV (LIG4)/XRCC4 complex, and RNAP II’s passage is provided by the elongation factor ELL. Based on [95,170].

**Figure 5:**
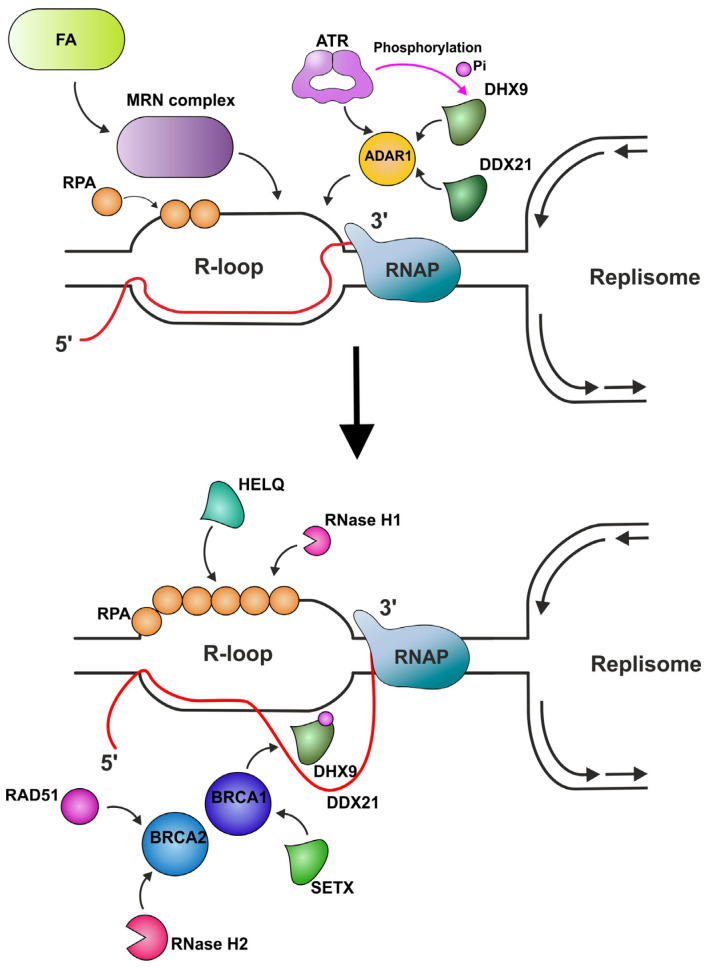
Several factors have a complex indirect influence on R-loop resolution. The MRE11-RAD50-NBS1 (MRN) complex suppresses R-loop formation through the recruitment of Fanconi anemia (FA) pathway complexes to these sites. Replication protein A (RPA) coats DNA-forming nucleoprotein filaments and signals to activate ataxia telangiectasia-mutated (ATM) and RAD3-related DNA damage response (DDR) kinases (ATRs), as well as attracting RNA-degrading RNase H1 and HELQ helicase to R-loops. ATR interacts with the RNA-editing enzyme ADAR1, which further recruits the specialized DHX9 and DDX21 helicases to unwind R-loops. ATR also phosphorylates DHX9, facilitating the interaction of this helicase with BRCA1 and RPA, and provides its association with R-loop sites. BRCA1 can associate with SETX helicase, thus unwinding R-loops. BRCA2 targets RAD51 to ssDNA rather than dsDNA, thus enabling RAD51 to displace RPA from ssDNA, and recruits RNase H2.

In contrast, the mechanisms that play a part in resolving R-loops, or preventing their formation, are intensively studied [102]. In general, the different factors protecting genomic stability from undesired R-loop propagation can be divided into three partially overlapping groups: (1) the factors preventing R-loop formation (Figure 2), (2) the factors resolving already formed R-loops (Figure 3 and Figure 4), and (3) the factors removing R-loops indirectly by restarting stalled replication forks and repairing DNA damage (Figure 5) [137]. The first group can be conditionally classified as all the factors that are responsible for DNA and chromatin structure stabilization (including topoisomerases, DNA helicases, and nucleosome assembly factors). The second group mainly consists of transcription and RNA metabolism factors (such as RNA-coating proteins, RNA helicases, and RNase H). Finally, the third group includes a range of protein factors that is involved in DNA metabolism and repair (such as the oncosuppressors BRCA1 and 2, the Fanconi anemia (FA) complex, ATR, and XPG) [137]. All the proteins that participate in different aspects of the protection of cells from R-loop propagation and are described in this review are listed in Table 2.

##### Factors Preventing R-Loop Formation

***RNA-coating proteins***

One of the possible ways for cells to prevent R-loop formation is thought to be the coating of nascent RNA with specialized proteins that are involved in processing and exporting newly synthetized RNA molecules [77,214], as was hypothesized for RPA for example [129]. In their recent work, Pan et al. demonstrated that the cohesins SA1 and SA2, the key components of the cohesin complex, play important roles in 3D chromatin organization, colocalizing with several R-loop sites (Figure 2) [141]. Considering the fact that SA1 and SA2 bind strongly to RNA, especially preferring DNA–RNA hybrids; this observation suggests that these proteins have some role in R-loop metabolism, although there is currently no direct evidence that they resolve R-loops.

***Chromatin structure***

There is also growing evidence that the chromatin structure itself may influence the prevention of pathological R-loop formation (Figure 2). Garcia-Pichardo et al. found several viable *S. cerevisiae* histone H3 and H4 mutants, characterized by increased levels of R-loops [215]. Additionally, Zhou et al. suggested that the loss of H3K9 dimethylation (H3K9me2), associated with transcriptional repression, provokes the accumulation of R-loops at the rDNA locus [216,217,218]. To ensure rescue from a situation in which TRCs can lead to the formation of stable R-loops and the stalling of replication forks, cells can switch between different types of chromatin modification, such as the change from crotonylation to the ubiquitination of H2A at Lys119 [219]. Thus, the H2AK119ub histone mark correlates with the repression of transcription [220] and was shown to promote the dissociation of RNAP II at reversive replication forks, preventing the transcription of genes located near the stalled replication forks, and thus preventing TRCs [219]. Recently, Bayona-Feliu et al. demonstrated that the depletion of the key chromatin factors INO80, SMARCA5, and MTA2 results in increases in TRCs, replication fork stalling, and R-loop-mediated DNA damage [142]. Overall, the association of various changes in histone functionality, leading to the disruption of genome heterochromatinization, with the increased accumulation of R-loops is in accordance with the tendency of more exposed DNA regions to favor DNA–RNA hybrid formation [221,222].

***Topoisomerases***

Given that the formation of R-loops requires the transcription process to occur and is facilitated by the negative supercoiled topology of DNA that is generated through the passage of RNAP, the prevention of R-loop formation can also be facilitated by the work of specialized topoisomerases. However, R-loop formation that is caused by the excessive presence of gyrase over that of Top1 leads to an increase in the hypernegative supercoiling of DNA [84,223]. It was shown that Top1, a ubiquitous member of the Type IB subfamily of topoisomerases, participates in preventing R-loop formation by resolving the accumulation of local negative supercoils on transcribed regions (Figure 2) [50,144]. Top1 is also required to prevent HO-TRCs by pausing the replication fork at the terminators of highly expressed genes containing R-loops [224]. Recently, it was demonstrated that a much-less-commonly studied member of the Type IA subfamily of topoisomerases, Topoisomerase III beta (TOP3B), also plays an important role in the suppression of R-loop accumulation [143,145,146]. Saha et al. proposed that TOP3B suppresses R-loop propagation in tandem with DDX5 helicase [225].

***PrimPol***

Among the different sequences that are prone to forming various non-B DNA secondary structures, a major proportion of the range of tandem repeats are also known as microsatellites [226]. For example, a long tract of polypurine–polypyrimidine (GAA)_n_ repeats can form H-DNA, a triplex non-B DNA structure, which is associated with the inherited neurodegenerative disorder Friedreich’s ataxia. This is very probably due to the tendency of such structures to efficiently block replication [151,227,228]. Moreover, such triplet tandem repeats are prone to R-loop formation [229]. It is interesting that considerably shorter repetitive tracts, which are distributed throughout the human genome [230,231], apparently provoke R-loop formation as well [151]. Šviković et al. demonstrated that the impediment of (GAA)_10_ repeat-induced R-loop formation and replication can be resolved by including PrimPol in the processive replication of this sequence [151]. The DNA polymerase called Primase-Polymerase (PrimPol) was identified over a decade ago and has been assigned with a role in DNA damage tolerance in eukaryotes [232,233,234]. Schiavone et al. demonstrated that PrimPol, although unable to directly replicate G-quadruplex structures, facilitates their bypassing by repriming downstream of these structures (Figure 2) [235]. These observations suggest that PrimPol has a role in TRC resolution.

##### Factors Resolving R-Loops

***RNase H and others***

The primary factors protecting genetic stability against the detrimental propagation of R-loops are currently thought to be RNA-cleaving enzymes, specifically RNase H (Figure 3) [236]. Monomeric RNase H1 and heterotrimeric RNase H2 are highly conserved among living organisms and were demonstrated to specifically degrade RNA in DNA–RNA hybrids [237,238]. The preference of these enzymes toward DNA–RNA hybrids is facilitated by their specialized hybrid-binding domain, and the RNase H domain ensures the degradation of RNA strands [237]. In *E. coli,* the interaction of RNase H1 with ssDNA-binding protein (SSB) binds RNase to DNA replication sites [239]. In eukaryotes, RPA has also been shown to be a key partner of RNase H1, not only colocalizing with this enzyme on R-loops, but also stimulating its nuclease activity [138,139]. RNase H1 and H2 both were demonstrated to disintegrate R-loops, but RNase H2 can also remove mis-incorporated ribonucleotides from DNA [153,155], as well as Okazaki primers during lagging-strand DNA synthesis [240]. 

It seems that RNase H2 plays a pivotal role in controlling co-transcriptional R-loop accumulation, as it has been shown that this nuclease binds directly to RNAP II (Figure 3) [154]. Nevertheless, Amon end Koshland demonstrated that an increase in the number of RAD52 foci, which is interpreted as a signal for R-loop-associated damage and homologue recombination (HR), is only observed in cases of diminished activity of both RNase H enzymes [204]. It was also demonstrated that another DNA repair factor, RAD52, plays an important role in R-loop suppression, as its deficiency causes R-loop accumulation, leading to DNA damage [205]. It is thought that RNase H1 is especially important for mitochondrial DNA replication and resolving R-loops [241,242]. In addition, RNase H1 and H2 seem to have different degrees of cell cycle dependence, allowing them to control R-loop accumulation under different conditions. RNase H2 appears to be restricted to the G2/M phase of the cell cycle, whereas RNase H1 is cell cycle-independent and is activated in response to high R-loop loads [243]. It was also demonstrated that *E. coli* RNase H1′s R-loop-degrading activity depends on the structural features of the particular R-loop in vitro, as well as the presence of RNAP [122]. It appears that RNAP can partially protect short R-loop segments from RNase H1-facilitated cleavage, but it is forced to dissociate if the length of the uncovered hybrid duplex segment is sufficient to initiate RNase H1 activity, at least based on the in vitro experimental results.

RNase H3, which is closely related to RNase H2 in its structure and found in some *Archaea* and bacteria [244], is also able to efficiently remove RNA strands from HO-TRC-induced R-loops [245].

With the details of the pathways of R-loop formation, signaling, and resolution being far from completely understood, new studies make various contributions to this field. One of the latest and most interesting propositions about the recruitment of RNase H1 to R-loop sites is that this occurs through RNA methylation facilitated by methyltransferase-like 3 (METTL3) [210]. *N*^6^-methyladenosine (m^6^A) RNA methylation is part of several important processes relating to RNA functioning. The establishment of this mark is facilitated by the methyltransferase complex of METTL3, METTL14, and the Wilms tumor 1-associated protein (WTAP), a mammalian splicing factor [209,211,212]. METTL3 is thought to have a special role in m^6^A RNA methylation in response to DNA damage [246]. Abakir et al. proposed that m^6^A RNA methylation plays a role in R-loop removal under physiological conditions [207]. Kang et al. added to this observation a potential mechanism for METTL3 enrollment into R-loop formation regions. They demonstrated that one of the transcriptional regulators, the tonicity-responsive enhancer-binding protein (TonEBP), recruits METTL3 for the m^6^A RNA methylation of RNA strands from an R-loop, and that this methylation, in turn, facilitates the recruitment of RNase H1 to degrade this R-loop [210]. In contrast, according to Hao et al., DDX21 helicase, which is involved in R-loop unwinding [178], can also attract METTL3 at R-loop sites for their further methylation and degradation [208].

A role in R-loop degradation was recently assigned to a different ribonuclease than RNase H type: DICER. Despite the fact that DICER ribonuclease is mostly known for its role in the formation of small regulatory RNAs and microRNAs in the cytoplasm [247,248,249], it can also function in the nucleus. It was demonstrated that DICER participates in resolving R-loops by specifically cleaving the RNA within R-loops [156].

Fairly recently, Lee et al. identified a novel RNA exonuclease 5 (*REXO5/LOC81691*) due to its elevated mRNA expression level in chronic myeloid leukemia patients [157]. This novel enzyme was proposed to degrade R-loops, as its knockout cells demonstrated elevated levels of R-loops and DNA damage. This suggestion was confirmed in latter experiments, in which the abilities of REXO5 to bind to R-loop structures and degrade RNA within R-loops were demonstrated.

Another interesting example of an enzyme which has shown itself to be a potential tool for RNA degradation in R-loops is flap endonuclease 1 (FEN1). FEN1 plays an important role in DNA lagging strand maturation, as well as in a long-patch variant of base excision repair (BER) [206,250,251]. It was recently demonstrated that FEN1, besides performing other essential functions facilitated through its RNA- and DNA-degrading activity, is able to process RNA to resolve R-loops [159,160]. Moreover, FEN1 was shown to be recruited to R-loops in cells, and this process was significantly fueled by oxidative DNA damage [158]. The role of the BER pathway in the resolution of R-loops and TRCs was also suggested in another recent work [252].

Additionally, a role in R-loop processing was suggested for another enzyme that is involved in DNA repair. Poly [ADP-ribose] polymerase 1 (PARP1) is an important part of several cellular processes besides DNA repair. This enzyme exhibits ADP-ribose transferase activity, catalyzing the transfer of ADP-ribose units onto itself, as well as other protein targets and DNA ends [253,254,255]. Laspata et al. demonstrated that PARP1 is not only able to bind R-loops in vitro, but also shows a tendency to accumulate at R-loop sites in cells [206]. Considering the important role of this protein in facilitating DNA repair, this observation suggests the involvement of new players in the task of dealing with R-loops.

***Helicases***

Although helicases are currently thought to play a secondary role in R-loop resolution compared with RNases H, a growing body of evidence is suggesting that different helicases are required for the efficient resolution of TRCs and to reduce the burden of R-loop accumulation in cells (Figure 3) [256]. Thus, it was shown that two *S. cerevisiae* Pif1 family DNA helicases, Pif1 and Rrm3, resolve R-loops at the tRNA gene (tDNA) regions [162], thereby preventing the arrest of the replisome at these genes during HO-TRCs [161].

A putative role in R-loop resolution is also attributed to a subfamily of proteins that contain a conserved DEAxQ-like domain with RNA/DNA helicase activity [257], namely senataxin (SETX) and Aquarius (AQR) proteins [91,107,258]. These proteins belong to the same helicase superfamily, SF1, which also includes Pif1 and RecQ-like family helicases [257,259]. First, it was shown by Mischo et al. that Sen1, an essential component of the Nrd1-Nab3-Sen1 (NRD) complex of *S. cerevisiae*, plays an important role in transcription-associated genetic instability, not only by participating in transcription termination [260], but also by restricting the accumulation of R-loops [258]. The importance of the human homolog of Sen1 (SETX) in resolving R-loops was also demonstrated [107,261]. SETX was shown to unwind R-loops in the promotors of the RNAP II-transcribed gene regions and is probably recruited to transcription pause sites through its interaction with BRCA1 [168,169]. The mechanism of resolution of TRCs via the helicase activity of SETX seems to be associated with the promotion of replication restart at R-loop formation sites through the MUS81–LIG4–ELL pathway, which involves the MUS81-mediated cleavage of the leading chain of the stalled fork, the DNA ligase IV (LIG4)/XRCC4 complex-facilitated religation of the fork, and RNAP II passage provided by the elongation factor ELL (Figure 4) [95,170]. It is worth noting that an MUS81-dependant mechanism of R-loop resolution was also demonstrated for DDX17 helicase [171]. Additionally, Zhao et al. demonstrated in their work that SETX acts redundantly with RNase H2, considering that the double deletion of these two enzymes leads to more severe consequences than the individual suppression of each of them [262].

The RecQ-like helicases family, which is named after *E. coli* RecQ [263,264], in humans is represented by five enzymes: RECQ1, BLM, WRN, RECQ4, and RECQ5 [265]. All the helicases in this family possess a DEAD/DEAH-box helicase-conserved C-terminal domain and can unwind a range of DNA structures, including G-quadruplexes and forked DNA duplexes [264]. It was recently shown that RECQ1, along with RECQ5, is involved in a multistep process of reactivating R-loop-induced stalled replication forks [95]. RECQ5 is known to be involved in the prevention of replication fork stalling on genes that are transcribed by RNAP I and RNAP II [150]. This helicase was demonstrated to play an important role in the resolution of TRCs, particularly by removing RAD51 from the stalled replication fork to facilitate MUS81 endonuclease’s cleavage of the fork (Figure 4) [95,147,148,149]. At the same time, the observation that in cells that are deficient in RECQ5, RAD18- and BRCA1-dependent RAD51 foci are accumulated, and that the localization of this accumulation indicates a rise in unresolved replication intermediates, indicates that RECQ5 may exert a significant effect on cells recovering from TRCs through putative mechanisms [150]. There is a possibility that RECQ5 works on G-quadruplex structures, as it was demonstrated that this helicase is able to destabilize such structures in vitro [266]. Another homolog of RECQ5, BLM, was shown to participate in R-loop suppression. It was demonstrated that BLM is not only able to unwind DNA–RNA hybrids in vitro [267], but also directly resolves R-loops in cells [164]. WRN, which is involved in the modulation of RNAP I/II-dependent gene transcription, was also demonstrated to participate in the suppression of R-loop accumulation [163,165,166,167].

One of the possible mechanisms for recruiting specialized helicases, namely DEAD/DEXH-box RNA helicases (such as DDX1, DDX5, DDX19, DDX21, DDX37, DDX50, and DHX9), to resolve undesirable R-loops is through the activity of the ATAD5 tumor suppressor [119,172,173,174,175,176,177,178]. ATAD5 plays an important role in maintaining genomic stability, mainly due to its function as a PCNA (the eukaryotic sliding clamp for replicative polymerases) unloader at the end of DNA synthesis during normal DNA replication [268,269,270]. Lee et al. demonstrated that ATAD5 increases the abundance of DEAD/DEXH-box RNA helicases at replication fork sites, thus participating in R-loop resolution [175].

Recently, it was demonstrated that the HELQ helicase belonging to the superfamily 2 (SF2) (unlike SETX and RECQ5, which are discussed above) is recruited to R-loops through its interaction with RPA [140]. It was shown to unwind R-loops in vitro as well as in cells, and more intriguingly to interact with nuclear 5’-to-3’ exoribonuclease XRN2, supposably coordinating the unwinding and degradation of R-loops.

A rather curious example of the enzymes that are involved in R-loop resolution is nucleolar N-acetyltransferase 10 (NAT10). This enzyme, besides its lysine and/or cytidine acetyltransferase activity, also harbors an RecD helicase domain (RHD) [179,180,181,182]. Su et al. demonstrated the ability of NAT10 to resolve R-loops in vitro by utilizing its RHD [182]. Moreover, the in vivo participation of NAT10 in controlling the propagation of R-loops was also dependent on its acetyltransferase activity. NAT10 was demonstrated to acetylate the DEAD-box RNA helicase DDX21 at K236 and K573, thus enhancing its helicase activity towards nucleolar R-loops [182]. It was found that NAT10 can facilitate R-loop resolution through different pathways, which highlights its role in protecting the cell from harmful TRCs.

##### Factors Providing Indirect R-Loop Removal

*DNA repair complexes*

Given that pathological R-loop propagation is clearly connected to the significant growth of DSBs [85,91,93], it is fair to suppose that some pathway cell activities addressing persistent R-loops are made possible by the proteins that are involved in DSB formation and repair. Indeed, it was demonstrated that the deletion of RAD50, an important member of the MRE11-RAD50-NBS1 (MRN) complex, results in increasing R-loop abundance in long coding genes [190]. The MRN complex is a highly conserved protein complex that plays an important role in the maintenance of genomic stability through its functions in early DNA damage signaling and the processing of DNA ends in the DSB repair pathway [191,192,193]. Chang et al. concluded in their study that the role of MRN in the suppression of R-loop formation at TRCs is provided through the recruitment of FA pathway complexes to these sites (Figure 5) and not through the nuclease activity of MRE11 [190,193]. This is in a good agreement with evidence showing that the homologues yeast complex MRX (Mre11-Rad50-Xrs2) is important for maintaining the stability of replisomes at TRCs with stalled replication forks in Sen1-deficient cells [271].

It seems natural that the types of cell that go through rapid proliferation will encounter TRCs more often. Thus, it was demonstrated that primordial germ cells (PGCs) are subjected to high levels of TRC-induced replication stress, while nevertheless preserving their genetic integrity with excellent efficiency [272]. It was discovered that one of the main factors for PGCs to cope effectively with TRCs is the presence of a functional replication-coupled FA pathway. FA proteins are represented by an FA core complex, the FANCI-FANCD2 complex, as well as downstream DNA repair proteins, including BRCA1 and 2, RAD51, XRCC2, and 9 [273]. Yang et al. showed that when the key step of FA pathway activation, the monoubiquitination of FANCD2, is disabled, rises in the number of stalled replication forks and R-loop accumulation occur [272,274]. This observation is in good agreement with the above statements on the role of BRCA1 and 2 in the suppression of pathological R-loop propagation.

*BRCA1* is a widely known tumor suppressor gene, whose protein product participates, through its interactions with different partners, in a variety of cellular processes, including DNA damage repair, replication fork protection, transcription, and cell cycle regulation [275,276,277]. It was demonstrated for mammalian and yeast cells that BRCA1 participates in transcription activation [278] and is able to interact with RNAP II through DHX9 [198,200]. The recent data has linked BRCA1 and R-loop resolution. It seems that BRCA1 participates in R-loop suppression during RNAP II stalling [195]. Moreover, it was shown that BRCA1 is able to associate with Sen1/SETX, thus probably facilitating R-loop resolution through this interaction (Figure 5) [194,196,197,199]. The connection between the role of BRCA1 (as well as BRCA2) in R-loop resolution and DHX9 helicase was highlighted in a recent study conducted by Patel et al., where deficiency in RNF168, an E3 ubiquitin ligase and a DSB responder that directly ubiquitylates DHX9, resulted in the accumulation of R-loops in BRCA1/2-deficient breast and ovarian cancer cells [279]. On the other hand, it was shown that BRCA1 participates in the activation of the persistent R-loop-induced HR pathway, involving this protein in the development of a negative cellular scenario that is caused by the formation of R-loops [280].

Although BRCA2 is also a known tumor suppressor protein, mutations of which play a critical role in the development of high-grade serous ovarian carcinoma, the mechanisms by which this protein contributes to early oncogenesis prevention continue to be debated [1]. Interestingly, a series of recent studies have linked this tumor suppressor to the transcriptional regulation and recruitment of RNase H2 to DNA damage sites (Figure 5) [202,203], as well as to the R-loop resolution process itself [1,201]. Goehring et al. demonstrated that the preferential occurrence of the newly fired replication origins (dormant origin) near the transcription termination sites of active genes during global replication stress leads to the accumulation of HO-TRCs in BRCA2-deficient cells [1].

***Transcription factors***

One of the most efficient strategies to deal with the detrimental consequences of HO-TRCs is to control the available amount of arrested RNAPs. It was first demonstrated that transcription termination factors could participate in TRC resolution through the observation that the chemical inhibition of the bacterial transcription termination factor Rho results in a rise in replication-associated DSBs [281]. Later, it was demonstrated that the depletion of Rho leads to persistent R-loop formation [213]. It was also shown that another transcription termination factor, Rat1/XRN2, facilitates transcription through R-loops, presumably by degrading an RNA strand with its 5’-3’-exonuclease activity and inducing the premature termination of arrested RNAP II [282,283]. The presence of bacterial transcription elongation factors, such as DskA, Mfd, and GreA/B, was also demonstrated to be important for resolving TRCs in vivo [284,285].

CDK12 was shown to be one of the key mediators of transcriptional elongation in eukaryotes, and recent papers revealed the connection between CDK12 and R-loops and TRCs [286,287]. It was demonstrated that the knockout of oncogene *CDK12* in murine prostate cancer cells leads to the increased formation of R-loops through the upregulation of the androgen receptor (AR) and its coactivator FOXA1 [288].

## 4. Conclusions and Future Perspectives

R-loops are ubiquitous structures that can be found in many living organisms; they are known to participate in a variety of processes, including DNA replication, the regulation of gene expression, and immunoglobulin class-switch recombination, but a possible imbalance in R-loop metabolism is recognized as a serious threat to genomic stability. In recent decades, HO-TRC-induced R-loop formation has received significant attention from the scientific community. Its role in detrimental events, such as replication fork disruption and a rise in DSB levels, as well as the resulting elevated mutagenesis, has been demonstrated in several studies. The normal level of R-loops in a cell is regulated by various factors and processes that control their formation and resolution [236]. However, little is known about the factors facilitating R-loop formation. Along with the existence of particular sequences in DNA, prone to forming R-loops, and the key role of RNAP pausing in TRCs, there is extensive evidence for the participation of certain protein factors, such as RAD51 and RAD52 [87,104], DHX9 [123], and PRC2 [124], in R-loop formation. However, even these scarce examples need to be further supported by new research, taking the existing contradictions in the available data into account.

As soon as the negative supercoiling of DNA is favorable for R-loop formation, Top1 relaxes this type of supercoiling, thus playing an important role in the prevention of R-loop formation [50,144,224]. The level of R-loops increases significantly if the functioning of factors that are responsible for the export of mRNAs, such as the THO/TREX complex, is disrupted in the cell [77,78,79,289]. If persistent R-loops are formed, RNase H1, 2, and 3 are the enzymes which specialize in the enzymatic degradation of these structures [154,241,242,243], although growing interest in this area of research is bearing fruit in the form of new candidates for this role [156,157,158]. On the other hand, R-loops can be resolved by specialized helicases that are recruited to transcription and replication machineries [55,162,258,290]. The important roles of numerous factors which are also involved in various DNA repair pathways and epigenetic methylation maintenance, also becomes undeniable [126,136,195,272,274]. There is growing evidence that it is the disruption of R-loop metabolism that subsequently leads to the replicative stress and genomic instability associated with such structures [55,291,292].

## Figures and Tables

**Table 2:** Protein factors involved in suppression of pathological R-loop accumulation.

Protein	Protein Function (Known Role in The Organism)	Role in R-Loop Suppression	Ref.
**Prevention of R-loop formation**			
**RPA**	Binds and stabilizes ssDNA intermediates that form during DNA replication or following DNA stress.	Protects the nascent RNA by coating it. Allows human DNA polymerases to initiate DNA synthesis utilizing RPA-generated R-loops, thus reproducing replication restart in vivo. Prevents R-loop-induced DSB formation in SETX-deficient cells. Attracts RNase H1 to the regions of the formed R-loops and enhances its nuclease activity. Recruits HELQ helicase to R-loops. Signals ATR activation.	[100,125,129,138,139,140]
**SA1, SA2**	Components of the cohesin complex that play an important role in 3D chromatin organization. Strongly bind to RNA, especially DNA–RNA hybrids.	Protect the nascent RNA by coating it.	[141]
**INO80**	ATP-ase of the chromatin remodeling complex.	Suppression of TRCs due to heterochromatinization.	[142]
**SMARCA5**	Helicase that possesses intrinsic ATP-dependent nucleosome-remodeling activity.		
**MTA2**	Acts as a component of the histone deacetylase NuRD complex, which participates in the remodeling of chromatin.		
**Fob1**	Nucleolar protein that binds to the rDNA replication fork barrier site. Required for replication fork blocking.	Suppression of TRCs due to replication blockage.	[43]
Topo IV *, DNA gyrase *, Top1 *, TOP1 TOP3B	Topoisomerases.	Prevention of topological stress accumulation in DNA.	[50,53,143,144,145,146]
T4 Dda *, Rep *, UvrD *, DinG *, PcrA *, Rrm3, Mfd *	Helicases.	Prevent TRCs from occurring by displacing proteins that may be an obstacle to the passage of the replication fork and RNAP from DNA. Mfd is an elongation factor that displaces RNAP from the replisome pathway.	[23,26,56,57,58,59]
**RECQ5**	Helicase.	Plays an important role in the resolution of TRCs, particularly by removing RAD51 from the stalled replication fork to facilitate MUS81 endonuclease’s cleavage of the fork.	[95,147,148,149,150]
**PrimPol**	DNA polymerase called Primase-Polymerase. Plays a role in DNA damage tolerance in eukaryotes.	The participation of PrimPol in the replication process of sequences that are prone to R-loop formation reduces their formation.	[151]
**XRN2**	5’-3’ exoribonuclease is implicated in transcription termination.	Supposably prevents formation of R-loop-degrading downstream RNA containing a 5′ monophosphate as part of the termination process for most RNAP II transcripts.	[152]
**Direct resolution of R-loops**			
**RNase H**	Endonucleases that specifically degrade RNA in DNA–RNA hybrids.	Direct nuclease digestion of R-loops. RNase H2 interacts with RNAP II.	[153,154,155]
**DICER**	Double-stranded RNA (dsRNA) endoribonuclease playing a central role in short dsRNA-mediated post-transcriptional gene silencing.	Specifically cleaving RNA within R-loops.	[156]
**REXO5**	RNA exonuclease.		[157]
**FEN1**	Structure-specific nuclease with 5′-flap endonuclease and 5′-3′ exonuclease activities involved in DNA replication and repair.		[158,159,160]
**Pif1, Rrm3**	Specific helicases.	Directly resolve R-loops and/or G-quadruplexes.	[161,162]
**BLM, WRN, RTEL1, PIF1**			[67,163,164,165,166,167]
**FANCJ**		Directly resolve R-loops through interacting with MutSβ and MLH1, the components of the mismatch repair complex MutLβ.	[8]
**SETX (Sen1)**	RNA/DNA helicase involved in diverse aspects of RNA metabolism and genomic integrity.	The mechanism of resolution of TRCs via the helicase activity of SETX seems to be associated with the promotion of replication restart at R-loop formation sites through the MUS81–LIG4–ELL pathway, involving MUS81-mediated cleavage of the leading chain of the stalled fork, DNA ligase IV (LIG4)/XRCC4 complex-facilitated religation of the fork, and RNAP II passage provided by the elongation factor ELL.	[95,168,169,170]
**DDX1**, **DDX5, DHX9, DDX17, DDX19, DDX21, DDX37, DDX50**	DEAD/DEXH-box RNA helicases.	Directly resolve R-loops.	[101,119,171,172,173,174,175,176,177,178]
**HELQ**	Helicase belonging to the superfamily 2 (SF2).	Unwinds R-loops in vitro as well as in cells. Interacts with nuclear 5’ to 3’ exoribonuclease, a transcription termination factor of Rat1/XRN2, and supposably coordinating the unwinding and degradation of R-loops.	[140]
**NAT10**	RNA cytidine acetyltransferase that catalyzes the modification of N_4_-acetylcytidine on mRNAs, 18S rRNA, and tRNAs.	Resolves R-loops through its RecD helicase domain activity. Also acetylates DDX21 at K236 and K573, thus enhancing its helicase activity towards nucleolar R-loops.	[179,180,181,182]
**Indirect impact on R-loop resolution**			
**ATAD5**	Tumor suppressor. Functions as a PCNA (the eukaryotic sliding clamp for replicative polymerases) unloader.	Increases the abundance of DEAD/DEXH-box RNA helicases at replication fork sites, thus participating in R-loop resolution.	[119,172,173,174,175,176,177,178]
**ATR**	Serine/threonine protein kinase which activates checkpoint signaling upon genotoxic stresses such as ionizing radiation, ultraviolet light, or DNA replication stalling, thereby acting as a DNA damage sensor.	Activated in response to HO-TRCs. Phosphorylates BRCA1, CHEK1, MCM2, RAD17, RBBP8, RPA2, SMC1, DHX9, and p53/TP53, which collectively inhibit DNA replication and mitosis and promotes DNA repair, recombination, and apoptosis. The phosphorylation of DHX9 by ATR facilitates its interaction with BRCA1 and RPA, leading to its accumulation at R-loops.	[100,121,183,184,185,186,187,188,189]
**ADAR1**	Catalyzes the hydrolytic deamination of adenosine to inosine in double-stranded RNA (dsRNA), referred to as A-to-I RNA editing.	Attracts ATR to R-loops through its interaction with TOPBP1 (scaffold protein that acts as a key protein–protein adapter in DNA replication and DNA repair and promotes the loading of RAD51). Is also suggested to attract DHX9 and DDX21 helicases to R-loops.	[101]
**MRN complex (MRE11-RAD50-NBS1)**	Plays an important role in early DNA damage signaling and the processing of DNA ends at DSBs.	Plays a role in suppressing R-loops through the recruitment of FA pathway complexes to these sites.	[190,191,192,193]
**BRCA1**	Tumor suppressor, participates in a variety of cellular processes, including DNA damage repair, replication fork protection, transcription, and cell cycle regulation, through its interactions with different partners. Participates in the FA pathway.	Participates in transcription activation and R-loop resolution and is able to interact with RNAP II through DHX9. Attracts SETX to R-loops. Was demonstrated to participate in the activation of the persistent R-loop-induced homologue recombination pathway.	[194,195,196,197,198,199,200]
**BRCA2**	Involved in double-strand break repair and homologous recombination. Participates in the FA pathway.	Acts by targeting RAD51 to ssDNA over dsDNA, enabling RAD51 to displace replication protein A (RPA) from ssDNA and stabilizing RAD51-ssDNA filaments by blocking ATP hydrolysis. Was also shown to recruit RNase H2.	[1,94,95,201,202,203]
**RAD52**	Involved in double-strand break repair. Plays a central role in genetic recombination and DNA repair by promoting the annealing of complementary ssDNA and stimulating RAD51 recombinase.	Signaling of R-loop-associated damage and homologous recombination.	[204,205]
**PARP1**	Poly-ADP-ribosyltransferase that mediates poly-ADP-ribosylation of proteins and plays a key role in DNA repair.	Exhibits a tendency to accumulate at R-loop sites in cells, possibly attracting other DNA repair enzymes.	[206]
**XPF** (**ERCC1**) and **XPG** (**ERCC5**)	NER endonucleases.	Attracted to R-loops and initiate DSBs at their ssDNA sites.	[136]
**METTL3**, **METTL14** and Wilms tumor 1-associated protein (**WTAP**)	*N*^6^-methyltransferase complex that methylates adenosine residues at the N_6_ position of some RNAs and regulates various processes, such as the circadian clock, response to DNA damage, and primary miRNA processing.	Attracts RNase H1 through m^6^A methylation of RNA. Possibly attracted to R-loops by DDX21 helicase.	[207,208,209,210,211,212]
Rho *	Transcription termination factor.	Prevents R-loop formation, possibly through transcriptional arrest.	[213]

* Procaryotic homolog.

## Abbreviations

The following abbreviations are used in this manuscript:

hm5C	5-hydroxymethylcytosine
m5C	5-methylcytosine
AQR	Aquarius protein
ATR	Ataxia telangiectasia-mutated (ATM) and RAD3-related DDR kinase
BER	Base excision repair
BLM	Bloom syndrome protein
BRCA1/2	Breast cancer type 1/2 susceptibility gene protein
CD-TRC	Co-directional TRC
DSBs	Double-strand breaks
dsDNA	Double-stranded DNA
dsRNA	Double-stranded RNA
GADD45A	DNA damage protein 45A
DDR	DNA damage response
LIG4	DNA ligase IV
DNMT	DNA methyltransferase
Erα	Estrogen receptor alpha
FA	Fanconi anemia
FEN1	Flap endonuclease 1
H3K9me2	H3K9 dimethylation
HO-TRC	Head-on TRC
HR	Homologous recombination
METTL3	Methyltransferase-like 3
MMR	Mismatch repair
m6A	N6-methyladenosine
NAT10	N-acetyltransferase 10
NER	Nucleotide excision repair
PARP1	Poly [ADP-ribose] polymerase 1
PRC2	Polycomb repressive complex 2
PCBP1	Polycytosine (poly(C))-binding protein 1
PGCs	Primordial germ cells
RHD	RecD helicase domain
RPA	Replication protein A
rDNA	Ribosomal DNA
RNAP	RNA polymerase
SETX	Senataxin
ssDNA	Single-stranded DNA
SSB	Single-stranded DNA-binding protein
SF2	Superfamily 2 helicases
TET	Ten-eleven translocation DNA dioxygenase
TonEBP	Tonicity-responsive enhancer-binding protein
Top1	Topoisomerase I
Top2	Topoisomerase II
TOP3B	Topoisomerase III beta
TC-NER	Transcription-coupled nucleotide excision repair
TRCs	Transcription–replication collisions/conflicts
TLS	Trans-lesion synthesis
WRN	Werner syndrome helicase
WTAP	Wilms tumor 1-associated protein
